# Improving Emotional Safety, Coping, and Resilience Among Women Conducting Research on Sexual and Domestic Violence and Abuse

**DOI:** 10.1177/08862605231207617

**Published:** 2023-10-24

**Authors:** Anjuli Kaul, Danai Daphine Chavendera, Katherine Saunders, Sharli Anne Paphitis

**Affiliations:** 1 King’s College London, London, UK

**Keywords:** vicarious trauma, violence exposure, domestic violence, sexual assault, mental health and violence

## Abstract

Secondary trauma arises through indirect exposure to trauma through engaging with first-hand accounts and narratives of traumatic events. While a significant amount of research has explored secondary trauma experienced by professionals who work with survivors of trauma, such as clinicians and front-line service providers, there is little research exploring the experiences of secondary trauma among violence researchers who routinely engage with traumatic first-hand accounts through their work. This study qualitatively explored violence researcher’s professional experiences of secondary trauma and their perceptions of what enables and constrains their own coping and resilience. Participants were recruited using purposive sampling methods. Semi-structured interviews were conducted online with seven female violence researchers from the United Kingdom. Questions explored participant’s experiences of secondary trauma symptoms related to their research, perceptions of their own coping and resilience, and experiences of organizational support that have enabled or constrained their resilience. Data were analyzed thematically using a coding framework applied reflexively across interview transcripts. All participants reported experiencing symptoms of secondary trauma from their work including cognitive disturbances; altered beliefs of themselves, others or the world; and challenges connecting with others. Participants’ assessment of their own expertise in violence research did not generally impact their perception of their own resilience. Organizational support for violence researchers was rarely provided and participants felt generally unsupported—left to manage any resultant distress alone. Research organizations and universities should implement trauma-informed policies which positively transform workplace culture, provide peer support spaces, and conduct effective training in order to mitigate psychological harm and promote resilience among violence researchers. Support should be tailored to the requirements of violence researchers, and institutions should develop policies that are specifically attentive to the needs of researchers who also have lived experience of violence and abuse.

## Introduction

The term “secondary trauma” describes the adverse impacts experienced by people who are indirectly exposed to traumatic material. Symptoms of secondary trauma may include cognitive disturbances, burnout, compassion fatigue, and emotional distress in the form of anger, fear, or intrusive thoughts (Devilly et al., 2009; Slattery & Goodman, 2009). Secondary trauma is conceptually similar to vicarious trauma in that they both cause the recipient mental and emotional harm following indirect exposure to trauma, but they have notable distinctions. Vicarious trauma is often used to describe a pervasive long-term disruption to an individual’s views of themselves and the world following prolonged and gradual exposure to other people’s reactions to trauma. On the other hand, secondary trauma describes a more acute onset of symptoms similar to that of Post Traumatic Stress Disorder (PTSD) (Guitar & Molinaro, 2017; Rauvola et al., 2019). A significant amount of research has explored secondary trauma by professionals who work with survivors of trauma such as front-line service providers (Ellis & Knight, 2021), domestic and sexual violence counselors (Jenkins et al., 2011), mental health practitioners (Cieslak et al., 2013), and therapists (Baum et al., 2014). However, there is comparatively little research exploring experiences of secondary trauma among researchers of domestic and sexual violence and abuse. People who research violence are frequently exposed to traumatic material through their work, including through hearing first-hand accounts and narratives of trauma when conducting interviews with perpetrators and survivors; recruiting participants in trauma services (e.g., Sexual Assault Referral Centers, prisons, women’s refuge centers, etc.); and absorbing distressing material through analyzing secondary data. Subsequently, violence researchers are susceptible to experiencing secondary trauma throughout their work (Grundlingh et al., 2017).

Domestic and sexual violence are known to affect women more commonly than men, with 31% of women globally having experienced some form of physical and/or sexual violence (Sardinha et al., 2022). Feminist theory highlights an association between the patriarchal systems, which place men in positions of structural power and the occurrence, and wide societal acceptance, of violence against women. Patriarchal societies uphold gender inequality through implementing fear at an ideological and structural level and impede female empowerment; this results in poorer mental health and well-being outcomes (Dobash & Dobash, 1983; Sinha et al., 2017). Consequently, female researchers may experience poorer mental health outcomes from the compounding effect of frequent exposure to gender-based violence material in the workplace and their own salient experiences as women existing in a patriarchal society. This hypothesis is consistent with existing research that suggests women are more susceptible to experiencing secondary trauma than men (Baum, 2016). Importantly, not everyone who is exposed to traumatic material goes on to experience secondary trauma (Ogińska-Bulik et al., 2021). A possible explanation for this experiential variance may be explained through the framework of constructivist self-development theory (CSDT) (McCann & Pearlman, 1990). CSDT suggests that there is no one standard response to traumatic material, and an individual’s response depends on (a) the features of the researchers’ work (e.g., the type of violence they research or their level of exposure to traumatic material), and (b) unique aspects of the researcher themselves (e.g., their sex, gender, or personal history). Both feminist theory and CSDT emphasize a holistic understanding of how a person may experience secondary trauma through the myriad individual and broader environmental factors that affect them.

Previous studies have used the CSDT framework to describe individual and organizational factors contributing to secondary trauma in domestic and sexual violence researchers. These factors have been summarized by The Sexual Violence Research Initiative (2015) in the context of a socio-ecological model. At an individual level, risk factors for secondary trauma include lack of support from friends, family, or colleagues; a personal history or prior exposure to violence, and young age or inexperience in the field of violence research (Bloom, 2003). Much of the literature on secondary trauma does not provide a standard definition or age range to describe the characteristic of inexperience. In this paper, we adapt the definition of an Early Career Researcher laid out by UK Research and Innovation (UKRI) to describe a researcher with low experience as someone within six years of their first academic appointment in the field of violence research (UK Research and Innovation, 2022). At the organizational level, risk factors for experiencing secondary trauma include workplace stigma around secondary trauma (Richardson, 2001), institutional tolerance and failures to respond to vicarious trauma (Rosenbloom et al., 1995), and lack of spaces provided to support self-care (Yassen, 1995). These factors implicate organizational structures as a target site for intervention against the harms that violence researchers may encounter through their work.

## Study Aims

The present study seeks to qualitatively explore domestic and sexual violence researchers’ experiences of these individual and organization level risk factors to understand how they impact emotional well-being and how organizations can prevent and reduce instances of secondary trauma. We aimed to answer two research questions:

1. How do researchers of violence and abuse experience secondary trauma and conceptualize resilience in the workplace?
2. How does the level of organizational support impact the level of self-reported mental health outcomes and experiences of secondary trauma in researchers of violence and abuse?

## Methods

### Ethical Approval

Ethical approval was obtained for the study (ethics clearance number: HR/DP-21/22-26497).

### Participants

Participants were recruited using purposive sampling methods. For inclusion in the study, participants were required to be (a) researchers of violence and abuse; (b) aged 18 years or over; (c) based in the United Kingdom; (d) members of the UKRI Violence, Abuse and Mental Health Network (VAMHN) (UKRI Violence Abuse and Mental Health Network, 2018). The VAMHN is a research network that aims to reduce the mental health problems associated with violence and abuse by connecting individuals and organizations who work in the field of domestic and/or sexual violence and mental health. The VAMHN gatekeeper was approached via email to obtain consent for recruitment and the study recruitment poster was subsequently shared with their ~1,200 members via their online newsletter and their >2,000 Twitter followers.

### Data Collection

A 31-item topic guide was developed for this project from the existing literature on secondary trauma (Supplemental Appendix A). The topic guide was created to guide the interviews toward relevant issues, including participants’ self-assessed level of expertise, their experiences of stress, symptoms of secondary trauma, organizational support, resilience, and personal coping strategies. A pilot interview was conducted with another researcher to test and evaluate the usability of the topic guide. The feedback from the pilot was used to refine the topic guide for use in the main study. Changes made to the topic guide included more targeted questioning around (a) the specific symptoms of secondary trauma and (b) the coping mechanisms utilized by participants.

The main study was comprised of semi-structured interviews conducted individually with participants over Microsoft Teams. Participants were given an information sheet to read prior to interviews containing full information on the study (Supplemental Appendix B). Interviews lasted up to 45 min and were carried out between March and June 2022. Interviews were recorded and transcribed verbatim.

### Data Analysis

All identifiable information about participants was omitted from the final transcripts. A thematic analysis of the interview transcripts was conducted. A coding framework was developed consisting of the most salient themes and concepts from the existing literature on violence research and secondary trauma (Supplemental Appendix C). The framework was applied reflexively to each transcript, being progressively amended and reapplied to earlier transcripts where necessary. All coding was conducted using NVivo 12 Pro (QSR International Pty Ltd., 2018). To ensure reliability and mitigate researcher bias, a second reviewer independently coded 50% of the transcript data and a 74.3% agreement rate was reached between the two coders. All discrepancies were resolved through discussion.

## Results

Seven violence researchers were recruited for inclusion in the study from five different universities in the United Kingdom. All participants were female and Caucasian. Participant ages ranged from approximately 25 to –65 years old. Table 1 shows defining information about each participant and their institution.

**Table 1.** Participant Characteristics.

Participant ID	Participant Age Range (years)	Length of Experience in Violence Research (years)	Institution ID
P1	25–35	3.5	A
P2	25–35	8	B
P3	25–35	3	C
P4	50–65	4	D
P5	25–35	1.5	A
P6	35–50	15	A
P7	30–40	10	E

### Expertise and Resilience

The mean number of years of experience was 6.43 (*SD*: 4.82; Range: 1.5–15.0). Self-reported expertise was generally high, with only P3 and P5 reporting that they were new to violence research. All but one participant (P2) considered themselves to be resilient, although most reported experiencing times of low resilience throughout their careers. Broadly, participants perceived an association between lower levels of expertise and poorer emotional outcomes in relation to their work. Participants reflected on their formative years in the workplace, describing this time as being particularly stressful and overwhelming. Participants with higher self-reported expertise credited their ability to successfully manage stress and access support to their increased experience and number of years working in the field of violence research.

Participants described the concept of resilience as the ability to process difficulties and overcome challenges. However, several participants expressed distaste for the term resilience, with the common narrative being that while one may appear resilient on the outside, in many cases they are simply masking challenging or difficult feelings in an effort to persevere and push through. Additionally, participants described resilience as a fluid and changing process that you have during some stages of your life but not in others, rather than what they saw as a common assumption that resilience is a constant state of being where one is either resilient all the time or not at all. There was an association drawn between broader organizational and personal systems and the ways they can support individuals to foster resilience. For instance, some participants found that social support promoted their resilience. One participant (P2) attributed their lack of resilience to “structural and social” factors, including a lack of initial high-quality emotional education at a young age, or access to feminist research principles earlier in their career. Overall, participants considered general discourse around resilience to focus primarily on an individual’s ability to cope, when in fact the responsibility of fostering resilience should be placed on the organization to create a supportive environment.

### The Workplace: General Attitudes and Contributors to Workplace Stress

Most participants ascribed any positive feelings they had toward work to the positive social impact of their research, particularly for survivors of violence and abuse. They felt that their work was meaningful and challenging in a way that made them feel fulfilled.

However, feelings of stress, isolation, and anxiety were often attributed to intrinsic aspects of the work, such as the need to constantly revisit traumatic material as part of the methodological process for literature review or data analyses. Additionally, the flexibility required to work with survivors, which included conducting interviews on evenings and weekends, resulted in some participants feeling overwhelmed, with less ability to create boundaries between their work and personal life. Participants found that the sensitive nature of their research meant they constantly managed their own emotions, leading to decreased productivity and exhaustion at having to develop and access their own coping mechanisms.

The general workplace culture was also identified as a contributor to stress. All participants were employed principally by universities, and described working in an environment where the general reaction to stress from colleagues was to “get on with it” with little acknowledgment of the traumatic nature of the work. This led one participant to describe feeling shame at having to take a career break in order to restore their resilience.

Feeling the burden of responsibility of doing right by survivors was also a concern described by some participants, who expressed being occasionally daunted by the complexities of researching violence and the many ways in which research can harm and re-traumatize survivors. An added layer of anxiety was described from their perceived responsibility to accurately share these survivors’ accounts.

P2: You kind of oscillate between feeling really like. . .hopeful and like you’re doing something useful and then feeling. Like. Crushed by the weight of the responsibility of kind of doing justice to the people that you’re- that you’re working with.

P6: So, I feel there’s a. A necessary commitment in doing this work to. . .accurately represent the views and experiences of survivors of abuse.

Participants further expressed frustration at their perceived limited ability to help the survivors with whom they work. This was often described in conjunction with concern from seeing harmful attitudes and actions being expressed toward survivors of violence, including from students and mental healthcare professionals.

P7: There’s only so much you can do as a researcher. . . [the impact of the work] is not always immediate or far-reaching.

### Experiences of Secondary Trauma

**Figure 1.**
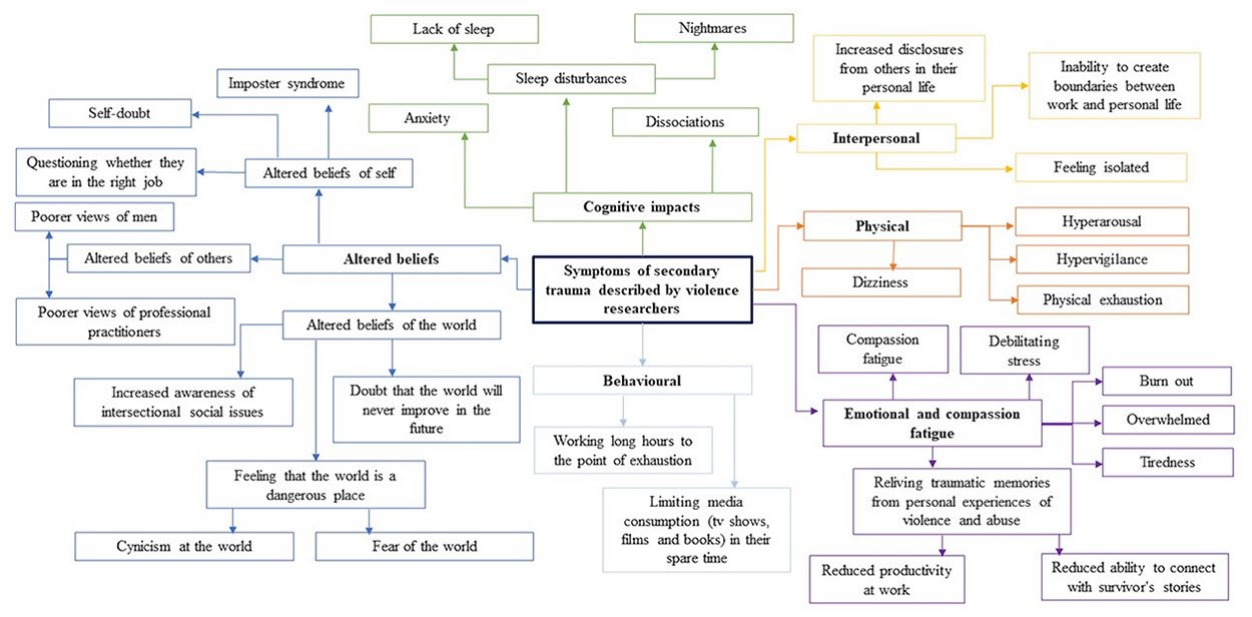
Concept map of the symptoms of secondary trauma described by violence researchers.

All participants reported experiencing symptoms of secondary trauma at some point in their careers. A concept map of the symptoms described by participants is shown in Figure 1.

#### Cognitive Impacts

Feelings of anxiety, isolation, and stress were commonly reported by participants. Some participants reported experiencing sleep disturbances and cognitive disturbances such as dissociations and memory impairments after hearing survivor’s accounts of violence and abuse.

P2: So, this wasn’t something that happened to me (. . .) But then, but yeah, just that realization that it sort of weaseled its way into my brain so much that I’d interpreted it as happening somewhere that I’d lived or something. It’s quite like. Traumatic.

#### Physical Impacts

Somatic symptoms of stress were often expressed and attributed directly to the work. Symptoms described included lethargy, dissociations, hypervigilance, exhaustion, and burnout.

P6: The sort of heaviness and tightness in my body. Feeling. A bit lethargic, potentially, maybe anxious?

P2: Well, I suppose the same as any kind of feeling of anxiety, just sort of shortness of breath and. . .Again, a kind of dizziness I think, also slightly dissociative and then, struggling to concentrate. Erm, and. . .and that’s I suppose when I’m- when I’m listening to people talk about their experiences. . .

#### Emotional Impacts and Compassion Fatigue

Compassion fatigue (i.e., feeling reduced empathy toward participants) was often described as a consequence of being emotionally drained and therefore being burnt out and unable to connect with survivors following consistent exposure to traumatic material. One participant mentioned experiencing compassion fatigue only toward the perpetrators of violence.

#### Interpersonal Impacts

Some participants reported the work had affected the way they interacted with others. They felt they could not fully discuss their work with other people in their lives due to the sensitive and potentially triggering nature of the research, leading them to feel less open and connected to others. Participants found that in instances when they shared information about their work, they were met with solemn reactions, or with more disclosures of violence and abuse, the latter of which would further confuse the boundaries between work and personal life.

P7: When you are a researcher who also teaches on these topics, students often feel like they can disclose to you. Uhm, which adds an additional level of. . .opportunity [for burnout] I suppose.

#### Altered Beliefs

Most participants described experiencing altered beliefs of themselves, others, or the world as a consequence of their work. Altered beliefs about oneself were often expressed as self-doubt, imposter syndrome, or questioning whether they were in the right job due to the emotional toll of their work. Many expressed altered beliefs about others, particularly toward men, due to the highly gendered nature of violence and abuse. Participants recounted how their work exposed them to issues around how survivors are treated by others, including the pathologizing of survivors’ trauma responses by healthcare professionals, and hearing students speak insensitively about issues around violence. This has affected participants’ views of the systems that support these groups.

Participants described feeling a sense of enlightenment around social paradigms such as the patriarchy and the disparity of privilege experienced by different groups in society. Participants also described more specific moments of realization throughout their career that the world was not a safe place, which manifested for some as cynicism or fear of the dangers one could experience.

### Impact of COVID-19

Participants described experiencing heightened cognitive impacts from their work during the COVID-19 pandemic lockdown, including anxiety, isolation, and burnout. Participants attributed these impacts to living and working in the same space each day, resulting in an increased difficulty in mentally separating from their work.

P6: At the beginning of [the COVID-19 lockdown] I found that I had very little division between work and home. I had a young son, so [. . .] I would have to stop for a period of time after five to [. . .] feed him, look after him, put him to bed. But when he was in bed, this is both me and my partner, we ended up working almost through the night [. . .] till midnight, going to sleep. And we’re in that cycle [. . .] and it was partly because our lives are so restricted at that point. We couldn’t go anywhere. [. . .] and I had to you know, for me, it culminated in the fact that I started to feel myself burning out.

However, one participant found that the COVID-19 lockdown did bring some positive changes, allowing them more time to manage their workload.

P4: Because COVID came along, my other work kind of died off for a while, so I had a lot of free time during lockdowns to do the research analysis.

Lockdown also had an impact on participant’s abilities to access their personal coping mechanisms. For instance, the restrictions imposed by lockdown meant that those who practiced outdoor exercise such as swimming, or socializing with friends were no longer able to utilize these approaches to improve their emotional well-being. Additionally, one participant who mentioned using food as an unhealthy coping mechanism found that this habit was exacerbated during lockdown, leading to increased negative perceptions about their own body.

P3: You get stressed, you eat and then eating makes you sad. Then you eat more ‘cause it makes you happy. And it’s that cycle. And I think that’s definitely something I’ve struggled with, particularly over the last. . .maybe two years? But I think COVID has impacted on that. That’s my kind of my main aspects. I know physically my body’s changed, which then makes me more stressed because of other things. But [. . .] especially now coming out of COVID, when you meet people and I- I have a little bit of a tub- a tummy now and people assume that I’m with child, and they’ll question like, “how do you do this type of research if you’re pregnant?!” and I’m not pregnant.

### Personal Characteristics

Participants often associated their experiences of secondary trauma with their personal characteristics, particularly their gender and their personal experiences of violence and abuse. Several participants reported experiences of violence and trauma in their own lives, or the lives of people close to them. Some reported having previously been triggered by survivor’s stories, which they related to their own experiences of violence.

P5: I started realizing that I probably experienced childhood trauma actually reading the material. Some of the trauma response that people exhibit [. . .] do resonate with me.

Participants also discussed how their experience of being a woman was linked, or seen by others as being linked, to the experience of violence at different levels. Some participants spoke of how their work in the field of violence research and exposure to media stories on violence against women may have sensitized them to the widespread prevalence of violence against women.

P4: This recent stuff with Sarah Everard, who hasn’t that. . .you know, what woman hasn’t that affected in the UK? So yeah, but I do feel more sensitive towards it.

Some also felt that their experiences being victimized as a woman helped them to better understand and relate to the survivors they worked with.

P6: As a woman, I have definitely experienced kind of the. . .some of the lower levels, sexual aggressions and maybe inappropriate sexual behavior [. . .] that you may not experience as a man.

Conversely, one participant spoke of how others assumed that her gender would hinder her ability to conduct violence research, specifically with perpetrators of violence.

P3: People have questioned how I have the ability to [talk to perpetrators of violence] as a woman. I find that really stressful as well. Of having to kind of justify that it doesn’t matter.

### Personal Coping Mechanisms

All participants reported using coping mechanisms to manage the emotional impact of their work. The most popular coping mechanism among participants was privately accessing professional mental health support, including private therapy and online Cognitive Behavioural Therapy (CBT) courses.

**Figure 2.**
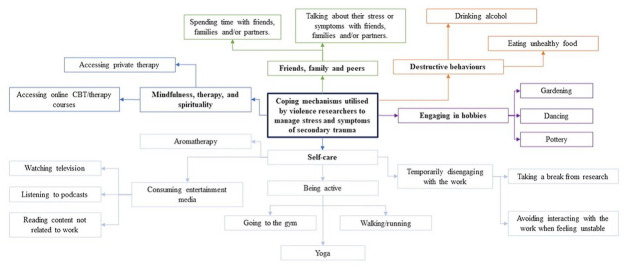
Concept map of the personal coping mechanisms utilized by violence researchers to manage stress and symptoms of secondary trauma.

Self-care and mindfulness were also common practices among participants. This encompassed a range of techniques and behaviors summarized in Figure 2. Participants also described the importance of these techniques in setting boundaries between their work and personal life.

P4: I can’t ignore those practices, ‘cause if I do, all the wheels come off and nothing’s gonna happen. . .And if I haven’t done my meditation, I start losing it in my head. So, I can’t do my research work. . .I’m really disciplined about this. But I have to be disciplined about it, ‘cause, if I’m not disciplined about it, I can’t think straight and I get dissociated and I get really upset and hysterical.

Destructive personal coping mechanisms were described by two participants. This included comments from P2 that they “could be drinking less,” and P3 who described coping using food in a way that was “not a healthy balance.”

### Experiences of Organizational Support

Although some researchers described feeling unsupported and isolated in their work at times, for many participants this was not due to a lack of supervisory support, which was often depicted positively, but due to a lack of peer support. Those who had experienced both strong supervisory and peer support expressed this as central to their well-being at work.

While some participants had accessed reflective supervision sessions at their organization, reliable and helpful methods of organizational support were not commonly described. The reflective supervision sessions mentioned were described as particularly helpful during lockdown, although it was noted that these were organized by a supervisor rather than their institution. Another participant found their organization’s drop-in sessions to be less helpful as they were not tailored toward violence researchers.

P3: They do have like drop-in sessions, but they are more open to everyone regardless of their research. And I haven’t been to that many just because you get people from all areas and it’s sometimes. . .I feel a bit lost because I’m trying to talk about something where you have to go back to basics and explain the whole rationale, and it’s just. . .it takes so much time and [. . .], well, why bother?

Counseling sessions were also available through some institutions; however, these were often described as inaccessible and not clearly signposted.

P7: I have-have lived experience of what I was researching and wasn’t really sure. . .at various times of the process where I could ask for support?

P2: Technically, my university offers counseling services, but they won’t. They’re not accessible to me because I have a therapist, so I can’t use those.

Most participants confirmed that their organizations did not provide any official training for their role as a violence researcher, and those who had accessed helpful training had received this previously for example, at previous jobs or in student-led groups. Topics addressed in these external courses which participants described positively included guidance on understanding one’s own emotions and stressors, as well as identifying ways to manage and mitigate stress. Some participants also described these training courses as providing a safe space to talk about their own emotions. The overall lack of organizational training and peer support networks were noted by participants as determinants of poor well-being among violence researchers, and indicative of a wider lack of support and acknowledgment of harms within the organization. Additionally, several participants said that they had raised these issues with their own organizations, and provided suggestions for improving researcher well-being, only to be ignored.

P1: Like I’m not going to sugar-coat it. I think all universities are extremely poor. They don’t recognize it. Like I said, I- I was trying to, you know, say to them you need to like to teach your students about this and they just kept ignoring my emails.

Overall, participants believed that supporting the emotional well-being of violence researchers was an institutional responsibility that was rarely actually met by the institution, with the responsibility usually falling on researchers themselves to foster support within teams and create their own coping mechanisms.

P2: I actually think that it should be less on the individual to kind of look after themselves and more on the structures and the team. Like to have a supportive team and to have, yeah a group with procedures and spaces in place for people to act. Rather than just saying, go and buy yourself a bottle of wine and have a bath.

### Suggestions for Organizational Improvement

The interview transcripts revealed five key areas for organizational improvement. These were (a) ethics, (b) funding, (c) training, (d) interpersonal and emotional support, and (e) policy. Suggestions from participants under each of these themes are presented in Table 2.

**Table 2.** Suggestions for Organizations to Foster Positive Well-being Outcomes in Violence Researchers.

Area for Organizational Improvement	Participants’ Suggestions for Organizational Improvement
Ethics	• Increased attentiveness and compassion from ethics boards around the potential harms of violence research on the researcher • Increased attentiveness from ethics boards toward the possibility of researchers having lived experience of violence and abuse, without imposing any pressure on them to disclose these experiences • Policies from ethics committees stipulating that all approved applications must outline the support in place to support the researcher’s emotional well-being • Willingness from ethics boards to provide more detailed guidance on issues around researcher safety in ethics applications
Funding	• Ringfenced budgets from funding bodies that reserve funds for researcher safety in grant calls • Ringfenced funding for researcher support in all grant applications • Building “emotional recovery time” for researchers into grant application project timelines so researchers have the time and space to process the emotional impacts of their work ○ Ringfenced budgets across organizations to set up and maintain ○ Peer support networks ○ Reflective practice/drop-in sessions ○ Trauma-informed counseling/therapeutic support services ○ Training courses for violence researchers • Departmental budgets to support researchers to attend external conferences/workshops/training which support their emotional well-being
Interpersonal and emotional support	• Strengthening team structures by creating and maintaining ○ Peer support networks specifically for violence researchers ○ Reflective practice/drop-in sessions specifically for violence researchers where principal investigators are not in attendance and researchers can discuss their challenges in a safe and free space ○ Trauma-informed counseling/therapeutic support services • Providing and signposting access to resources specifically for violence researchers around what secondary trauma is, how to manage difficult emotions, and how to access support • Signposting access to external violence research peer support networks • Buddy systems set up by the organization to allow junior researchers to form connections and learn about the realities of the work from more experienced researcher • Fostering a culture of acceptance and support at the organizational level by ○ Encouraging time off/flexibility to allow researchers time to support their emotional well-being ○ Offering “mental health days” where researchers can take time off to support their mental health without having to tell anyone their reasons ○ Circulating resources on what secondary trauma is and how to identify mental health problems in yourself to make it less taboo • Transparency from organizations around where support can be accessed and the nature of this support for example, group vs. individual, how many sessions are available, etc. • Sharing resources around what trauma-informed approaches are and how to apply them to research
Policy	• Creating policies for organizations to acknowledge and display awareness of researcher trauma, and clearly outlining their commitment to reducing this • Developing formal policies and procedures that outline clearly how researchers may access emotional support through their organization • Developing formal codes of conduct for any peer groups or reflective drop-in sessions to outline acceptable behavior in these groups and to prevent harmful behavior from occurring within these structures • Policies outlining the purpose and importance of taking well-being leave • Developing risk assessment protocols for violence researchers to account for the possible harms they may encounter
Training	• Providing trauma-informed training courses and training resources designed specifically for violence researchers on topics such as: ○ How to recognize secondary trauma symptoms ○ How to manage emotional stressors associated with their work ○ How to conduct trauma-informed research with the researcher’s emotional safety in mind ○ How survivor researchers may support their emotional well-being ○ Time management and workload prioritization to mitigate burnout • Ensuring all training and resources operationalize feminist principles

#### Ethics

Participants viewed attempts by ethics committees to address researcher safety as tokenistic, without consideration of the practical implications of protecting one’s mental health when conducting violence research. As a result, participant suggestions for improvement primarily revolved around an increased understanding and attentiveness to researcher safety and the needs of survivor researchers.

P7: There are, you know, these wildly ethical. . .systems to get approval to ensure no harm is done to a participant. Uh, and often if you are asked what you’re doing to prevent harm to yourself, a very cursory, “I will debrief with my supervisor” or “I will. . .,” you know, seems to be enough. Doesn’t appear to be a more preventative or. . . holistic approach.

#### Funding

Participants expressed frustration at the lack of funding leveled at maintaining researcher safety. A common suggestion to tackle this included ringfencing funds for researchers to access emotional support. One participant noted that many external conferences and skills workshops are available to teach helpful approaches to managing researcher stress, but they are often too expensive to be considered accessible.

#### Training

Most participants highlighted the need for all resources, training, and support services provided by an organization to be both trauma-informed and specifically tailored toward the needs of violence researchers.

#### Interpersonal and Emotional Support

A common suggestion for improving organizational support was for institutions to establish and maintain peer support networks with the recognition that violence researchers in an organization may be scattered across multiple departments or campuses. Organizations could also clearly signpost external networks and communities of violence researchers. Overall, there was a strong emphasis on the need for organizations to create dedicated space for violence researchers to safely discuss work-related issues and challenges in a group environment.

P2: I think it might be really helpful if my research team hosted like a weekly, fortnightly kind of drop-in session that was necessary without the PI, the boss. Erm, just to all talk about what we’ve been finding difficult.

P6: Yes, formal supports are helpful, but it’s also that peer-to-peer support that’s really incredibly valuable as well, and some people may not feel comfortable with this kind of formal based supports

#### Policy

Participants frequently expressed the belief that their university did not fully understand or acknowledge the harmful effects and complex emotional difficulties they experienced as a result of working in violence research. Strengthening internal organizational policies was frequently suggested by participants, not only to acknowledge the harmful nature of the work at an organizational level but also to clearly outline the organization’s specific commitments to supporting the well-being of violence researchers.

## Discussion

Symptoms of secondary trauma were widely reported by participants, indicating negative mental health outcomes are commonplace among violence researchers. Feelings of anxiety, isolation, and blurred boundaries between work and personal life were among the most commonly reported symptoms.

Over half the participants reported lived experience of violence and abuse and noted times when they felt triggered when hearing other survivors’ accounts. This may be a common experience among violence researchers, where there is likely a high proportion of survivor researchers who may feel a personal motivation to become involved in the field (Williamson et al., 2020). Additionally, researchers are at an increased risk of experiencing secondary trauma if they have a personal history of trauma themselves (Bloom, 2003) and a personal history of psychological problems (Hensel et al., 2015), the latter of which is also associated with experiencing violence and abuse (Trevillion et al., 2012). It is therefore likely that survivor researchers have an increased vulnerability to experiencing symptoms of secondary trauma. Negative coping styles and inexperience are also associated with secondary trauma (Kulkarni et al., 2013), a relationship explored by the participants with high levels of expertise, who described feeling more resilient with age as they had more time to identify and manage ways of coping with the difficulties of the job. Younger and more inexperienced researchers may experience more secondary distress as they have not yet developed protective mechanisms to cope with the traumatic material they encounter through their work (Bell et al., 2003). However, the singular participant who reported having low resilience had over 8 years of experience in the field and also engaged in negative coping styles, indicating a more complex interaction between work-related factors (such as expertise and access to support) and personal factors (such as age and sex).

Beyond individual factors, participants generally attributed their negative mental health outcomes to the repetitive and isolating nature of the work, exposure to survivors’ first-hand testimonies and the challenges of operating within the academic workplace. The latter encompassed the culture of productivity and the pressure to rapidly release outputs within academia, which was a hindrance to participants’ taking time off, and a contributor to feelings of shame and burnout. This experience has previously been described as a consequence of the “weight of positivism in academia” which suppresses the researcher’s ability to engage with their own feelings when their primary concern is collecting data with highly distressed participants (Markowitz, 2021, p. 94). Relatedly, some participants recalled disclosing their experiences of poor mental health to colleagues and being met with dismissive or minimizing responses. (Williamson, 2020, p. 56) hypothesizes that this cultural attitude may in part be due to the traditional view that academic research is “objective, detached and neutral” whereby researchers are not supposed to have any emotional responses to their work.

Despite poor mental health outcomes being common among violence researchers, participants felt that their organizations did not acknowledge or sufficiently support them with managing the emotional harms they experienced from their work, being left instead to utilize their own personal coping mechanisms. This was particularly demonstrated when exploring narratives around resilience, which was described negatively by some participants who felt the term placed responsibility on the individual to cope with emotional difficulties alone. Instead, they felt that fostering resilience should be the primary responsibility of their organizations and that most organizations were currently failing to support violence researcher well-being.

Our findings demonstrate that violence researchers would benefit from the organizational provision of peer support spaces, which participants with both high and low expertise valued indicating that all researchers, regardless of seniority, would benefit from these networks. Furthermore, we found that violence researchers had a strong desire for trauma-informed training on managing the emotional challenges of their research. Training should provide specific attention to the interplay of lived experience and secondary trauma which should guide violence researchers on how to navigate the experiential intersection between the personal and the professional. This attentiveness could manifest in the provision of spaces during training for researchers to discuss their lived experiences in a safe and private space (Ellsberg & Heise, 2002). Additionally, participants felt their research had sparked a “feminist consciousness” within them (Kiernan, 2016), where they gained an increased awareness of the damages of patriarchal norms and structures, which led to altered views of men. One participant specifically hypothesized that they would have been more resilient had they had been introduced to feminist principles earlier on in life, indicating training resources could incorporate feminist principles to mitigate these altered views. Further suggestions around improved attentiveness to researcher safety from Ethics Committees have previously been identified in the literature (Mckenzie et al., 2017), but participants from the current study felt that even when Ethics Committees did include items on researcher safety, it often felt tokenistic, and they were left wanting more appreciation of the emotional harms posed during violence research.

Beyond the improvements suggested by participants for research institutions, there was a fundamental desire for organizations to recognize and acknowledge the emotional distress experienced as a result of working in violence research, whether through the ethics process, the provision of training, or organizational policies. This need for acknowledgment may stem from a belief that their organizations do not prioritize their emotional well-being.

### Strengths and Limitations

This study addresses the dearth of research exploring the experiences of secondary trauma and organizational support in violence researchers, a typically under-researched sample. Additionally, while much of the literature around secondary trauma focuses on individual risk factors, we identify practical recommendations at the organizational level that can be feasibly implemented by academic institutions to improve the well-being of violence researchers under their employment from the perspective of violence researchers themselves. However, the present study does have some limitations.

This study had a small sample size, which may be attributed to the limited study timeline which stipulated a short, 1-month recruitment phase. All participants were Caucasian and female. Violence and abuse is an intersectional issue affecting different genders, races, and ethnicities differently, future research should ensure that these perspectives, beliefs, and experiences around secondary trauma are explored. Additionally, the international generalizability of our findings is limited as all participants were based in the United Kingdom, and so may not capture the barriers and access to support experienced by violence researchers in other countries. Additionally, all participants in the current study were academic researchers who worked in a university, therefore excluding perspectives from other work environments.

### Implications

Our findings indicate that research organizations and universities must do more to improve mental health outcomes in violence researchers through workplace culture reform and providing trauma-informed training and support. Universities should implement policies that foster a culture of acceptance and empathy toward violence researchers to mitigate burnout. Studies have shown that academic researchers work more overtime than other professions (Fontinha et al., 2019), and given the traumatizing material that violence researchers engage with, universities should actively encourage time off and breaks away from work. Universities should also work to provide and properly signpost peer support networks and training which are specifically tailored toward violence researchers and attentive to personal experiences of violence and abuse.

Future research may wish to compare violence researchers’ experiences of different types of macro-level support to find which are the more effective methods of promoting researcher well-being. Future studies may also address whether researchers with lived experience of violence and abuse have different needs to effectively reduce secondary trauma symptoms than those who do not have lived experience. It may also be beneficial to capture the experiences of violence researchers in independent research organizations or charities to identify how experiences differ across sectors.

## Conclusion

Symptoms of secondary trauma are commonly experienced by violence researchers. Mental harms arise from a range of sources, including the traumatic nature of the research, workplace stressors, and insufficient support and resources provided at an organizational level. Academic institutions should ensure that funding and space is ringfenced to foster the development of violence-specific peer support spaces and the provision of trauma-informed resources and training. Internal policies and ethics committees should also work to address and mitigate the negative mental health outcomes experienced by violence researchers in the workplace.

## Supplemental Material

sj-docx-1-jiv-10.1177_08862605231207617 – Supplemental material for Improving Emotional Safety, Coping, and Resilience Among Women Conducting Research on Sexual and Domestic Violence and Abuse Supplemental material, sj-docx-1-jiv-10.1177_08862605231207617 for Improving Emotional Safety, Coping, and Resilience Among Women Conducting Research on Sexual and Domestic Violence and Abuse by Anjuli Kaul, Danai Daphine Chavendera, Katherine Saunders and Sharli Anne Paphitis in Journal of Interpersonal Violence

sj-docx-2-jiv-10.1177_08862605231207617 – Supplemental material for Improving Emotional Safety, Coping, and Resilience Among Women Conducting Research on Sexual and Domestic Violence and Abuse Supplemental material, sj-docx-2-jiv-10.1177_08862605231207617 for Improving Emotional Safety, Coping, and Resilience Among Women Conducting Research on Sexual and Domestic Violence and Abuse by Anjuli Kaul, Danai Daphine Chavendera, Katherine Saunders and Sharli Anne Paphitis in Journal of Interpersonal Violence

sj-docx-3-jiv-10.1177_08862605231207617 – Supplemental material for Improving Emotional Safety, Coping, and Resilience Among Women Conducting Research on Sexual and Domestic Violence and Abuse Supplemental material, sj-docx-3-jiv-10.1177_08862605231207617 for Improving Emotional Safety, Coping, and Resilience Among Women Conducting Research on Sexual and Domestic Violence and Abuse by Anjuli Kaul, Danai Daphine Chavendera, Katherine Saunders and Sharli Anne Paphitis in Journal of Interpersonal Violence
