# Supplementary figures and images for: Improving Emotional Safety, Coping, and Resilience Among Women Conducting Research on Sexual and Domestic Violence and Abuse
Source: J Interpers Violence. 2023 Oct 24;39(5-6):1327–50. doi: 10.1177/08862605231207617 (PMC10858617; doi:10.1177/08862605231207617)

# **Appendix A. Participant Information Sheet**

**
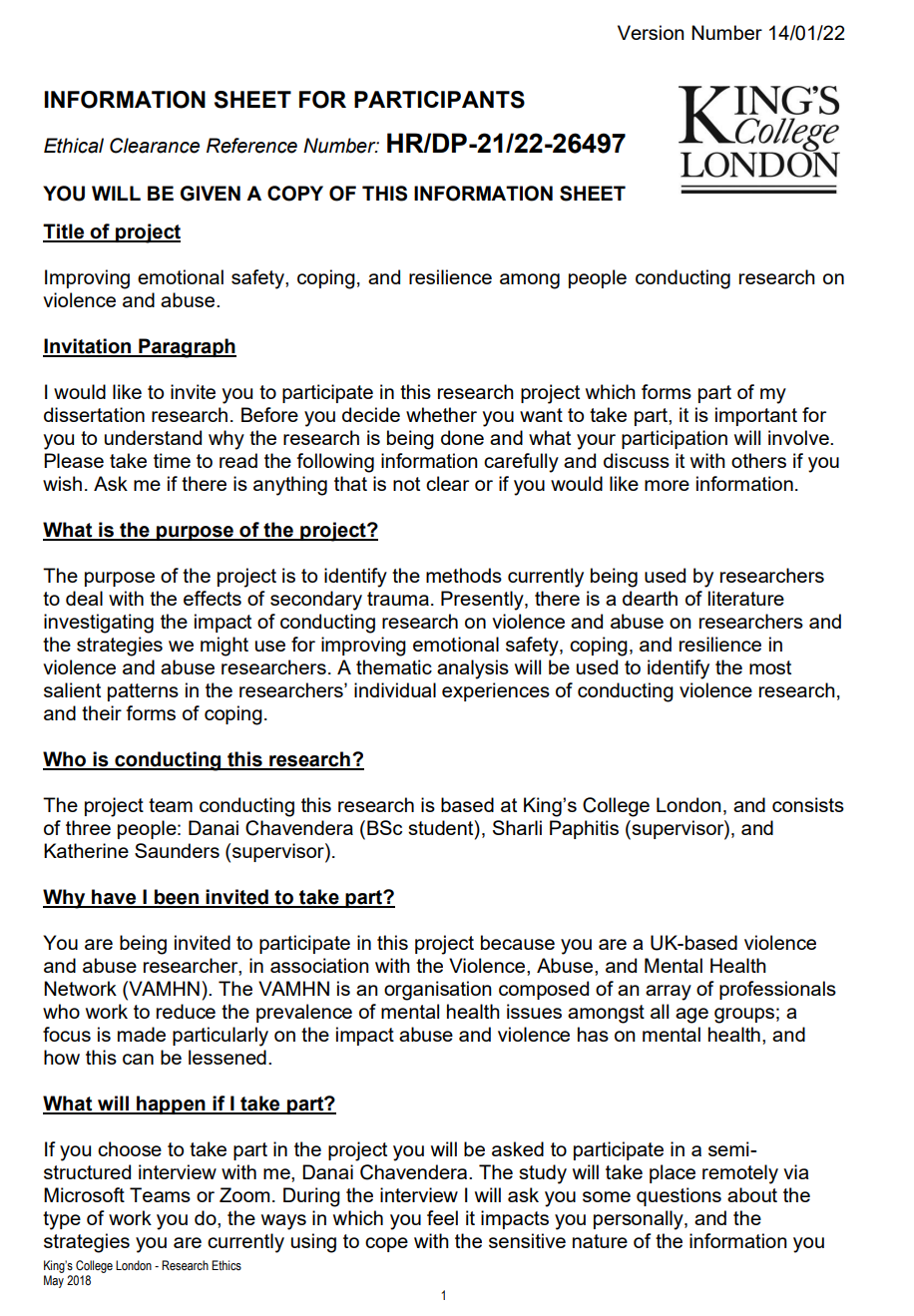
**


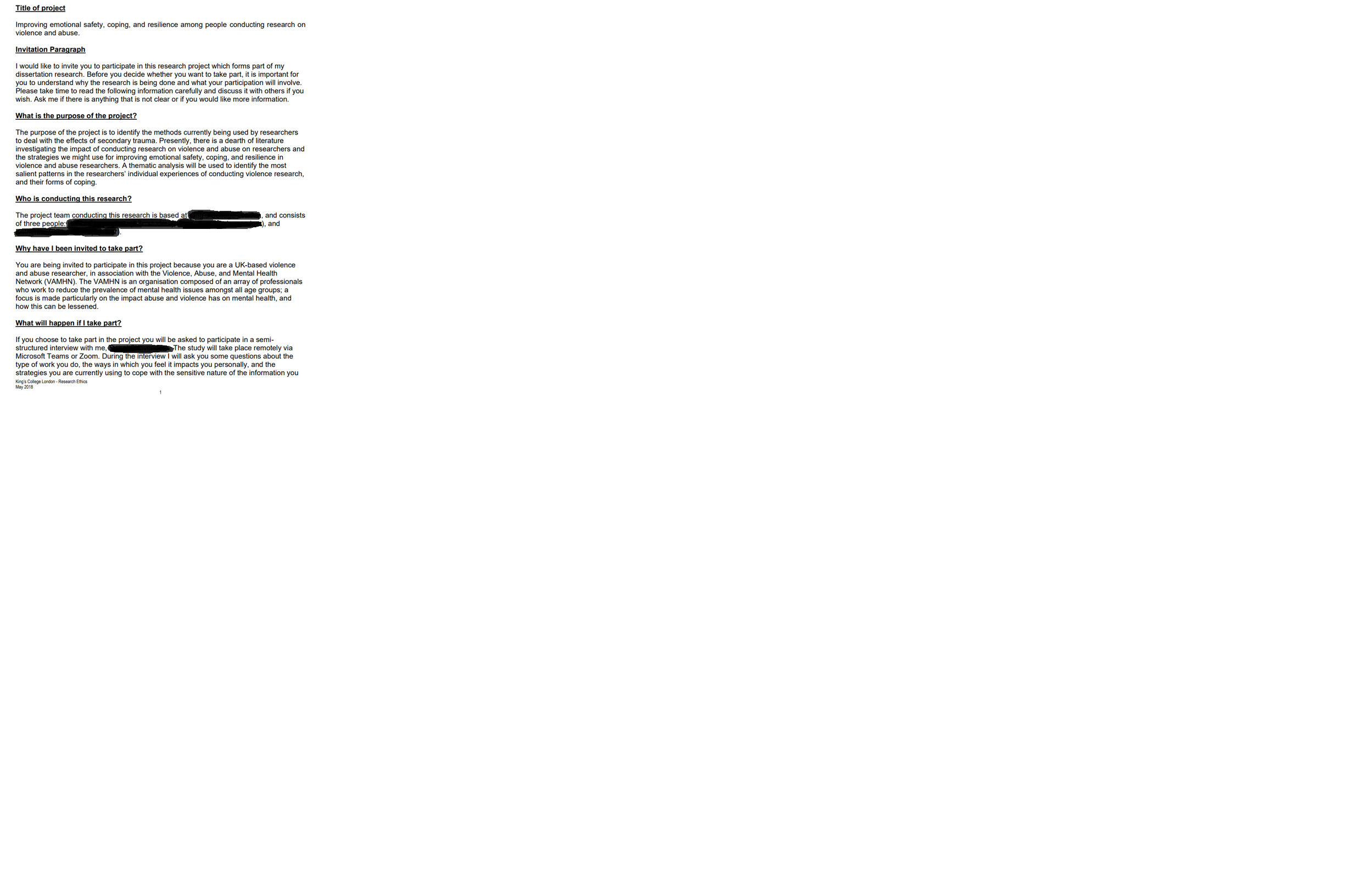


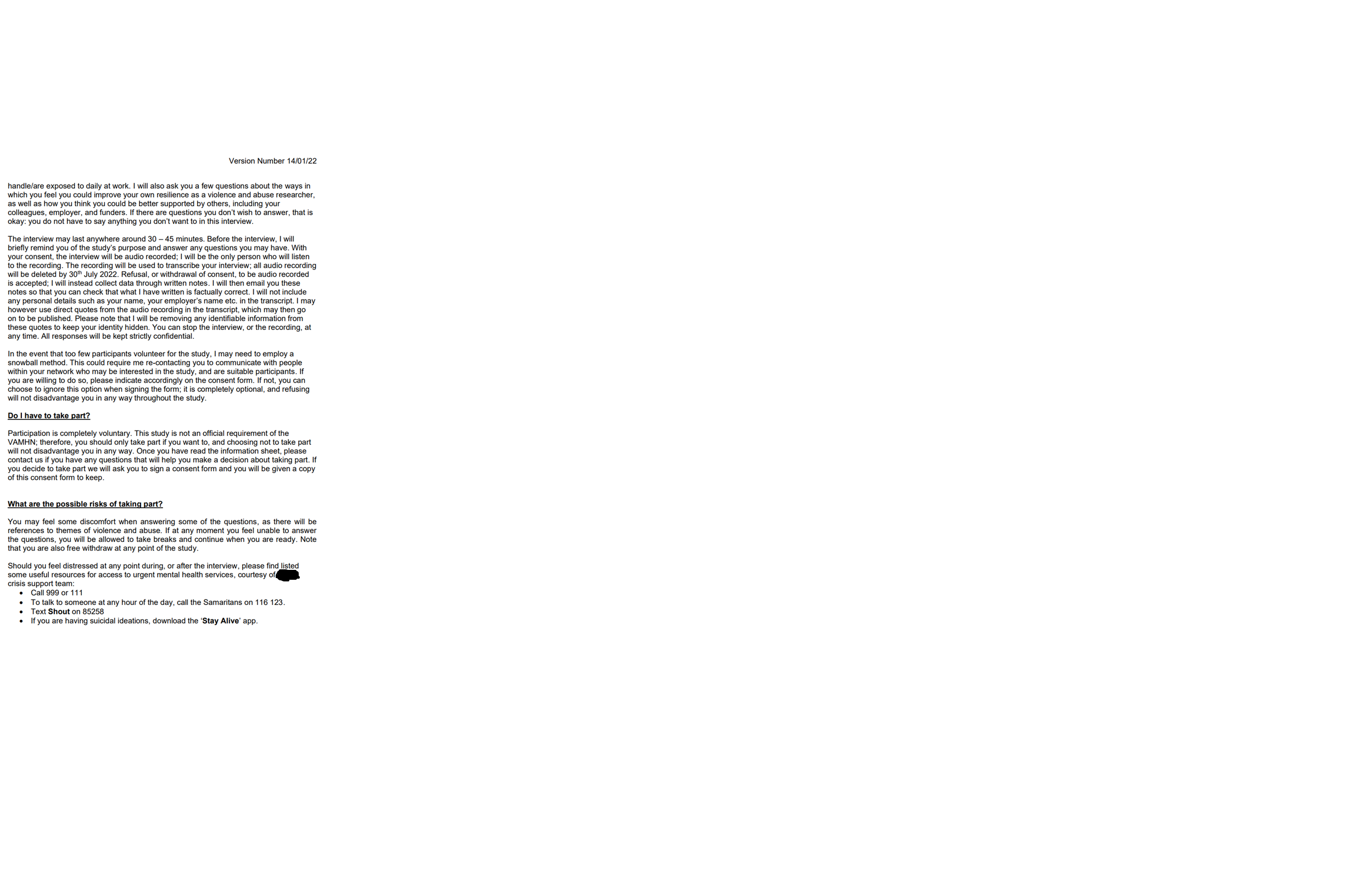


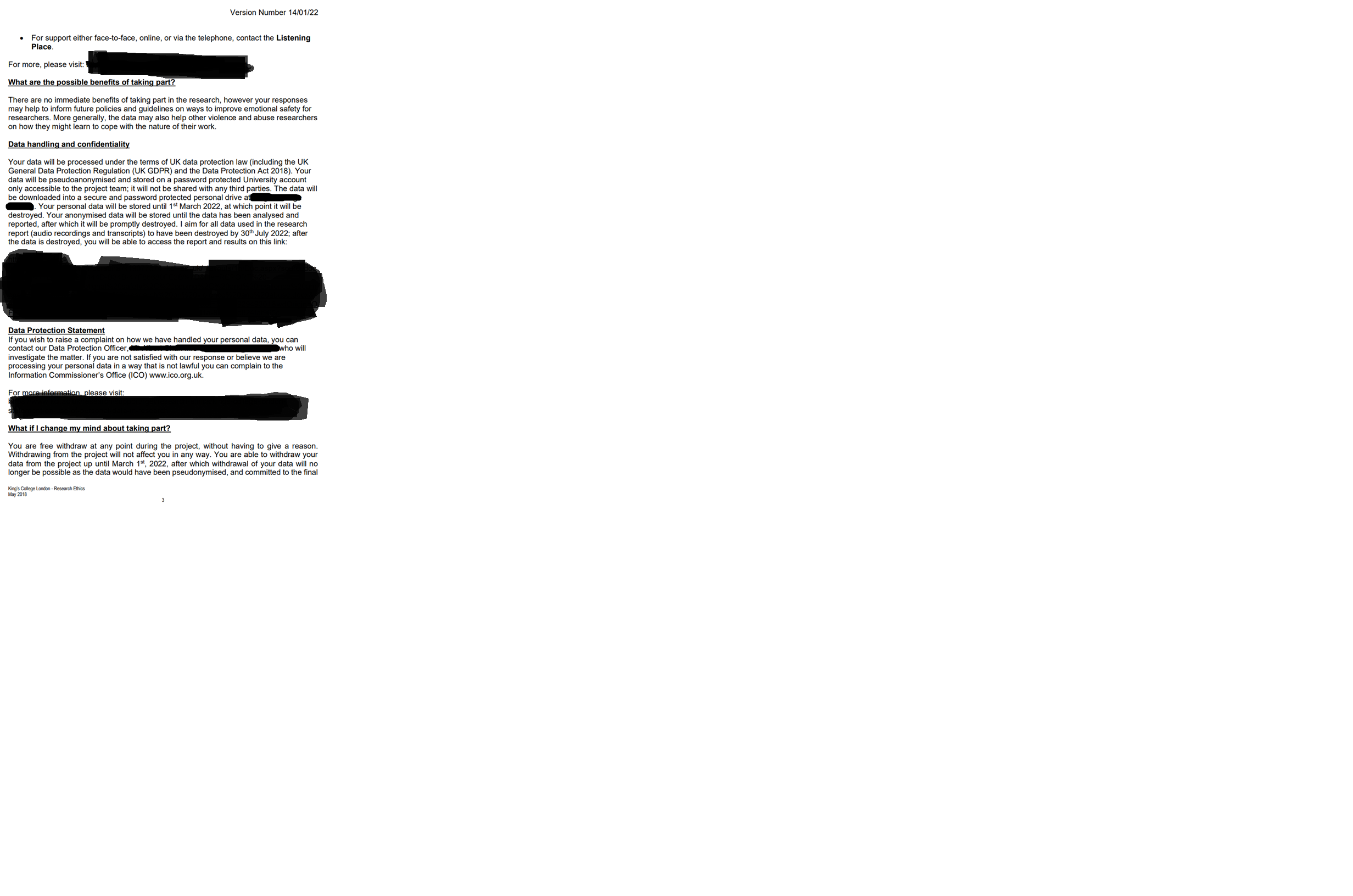


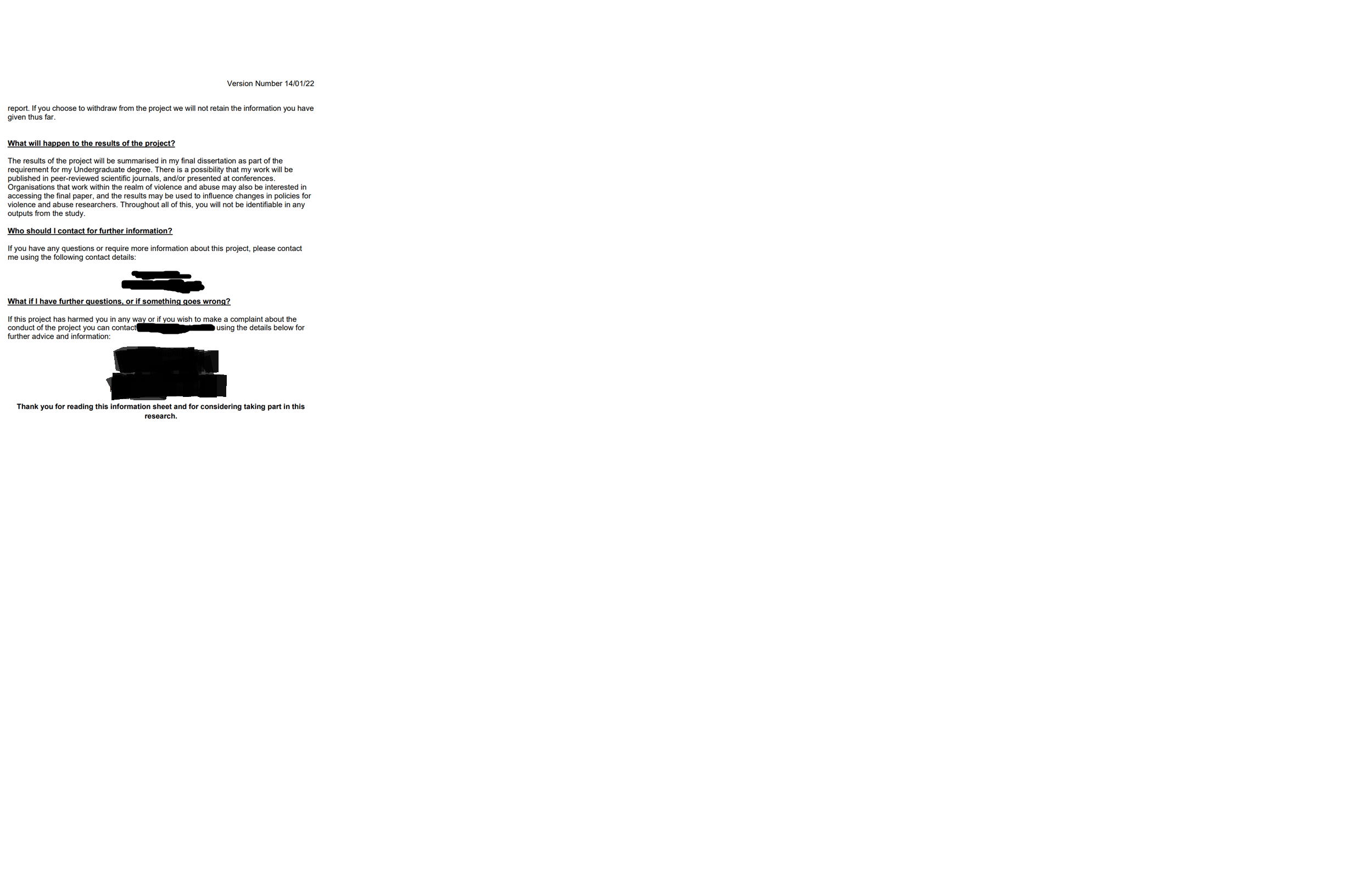

Supplement: sj-docx-1-jiv-10.1177_08862605231207617 – Supplemental material for Improving Emotional Safety, Coping, and Resilience Among Women Conducting Research on Sexual and Domestic Violence and Abuse [file sj-docx-1-jiv-10.1177_08862605231207617.docx]
