# Supplementary material for: Improving Emotional Safety, Coping, and Resilience Among Women Conducting Research on Sexual and Domestic Violence and Abuse
Source: J Interpers Violence. 2023 Oct 24;39(5-6):1327–50. doi: 10.1177/08862605231207617 (PMC10858617; doi:10.1177/08862605231207617)
Supplement: sj-docx-2-jiv-10.1177_08862605231207617 – Supplemental material for Improving Emotional Safety, Coping, and Resilience Among Women Conducting Research on Sexual and Domestic Violence and Abuse [file sj-docx-2-jiv-10.1177_08862605231207617.docx]

# **Appendix B. Interview Topic Guide**

| **1. Introductions and Background**  1.1. Introductions  Hello, my name is X, and I will be interviewing you today.  How are you doing?  1.2 About the project  You saw the advert and expressed your interest in taking part in this study about improving the emotional safety, resilience, and coping strategies for violence and abuse researchers. Can you confirm that you have read and understood the participant information sheet (PIS)?  1.3. Discussing the consent form and gaining verbal consent to continue to the interview questions  Also, as stated in the PIS, this conversation will be audio recorded. Is that okay with you, and do you still wish to continue?  This interview should not take more than 45 mins – are you ready to begin?  **2. Background**  2.1. Easing into the topics  Q1. How would you describe the type of research you conduct, and how long have you been this type of researcher?   - Can you describe your work/caseload, and do you have feel like you have a sense of control over it? - Do you usually work alone, or in teams? Roughly how many people in this team?   Q2. Generally, how does this type of research make you feel?   - Is this the same way you felt when you started doing this kind of research? (extra/prompt) - Do you think this has affected your sensitivity to violence in any way? This could be an increase/decrease/or no change at all   **3. Stress and Secondary Trauma**  3.1. Stress  Q3. In your own words, how would you describe the term ‘stress’?  Q4. And using your definition, would you describe your role or research work as ‘stressful’?  In what ways/How so?   - How often does your work leave you feeling this way? (extra/prompt)   3.2. Secondary trauma  Q5. An area of literature that has growing interest is that of secondary trauma - are you familiar with this term?  \|_ The act of an individual becoming indirectly traumatised as a result of being exposed to first-hand accounts of traumatic material.  Q6. Do you think that you have ever experienced secondary trauma as a result of the research work you do? If so, can you describe any specific symptoms that you may have felt.   - Do you still deal with any of these issues? - Speaking at a deeper level, is there anything in your personal history (e.g., something that you have ever experienced or witnessed in your life) that could be affecting how you respond to the traumatic material at work? - Also, previously you mentioned that you have a relatively HIGH/LOW caseload – do you think that this is this a relevant factor in terms of your response to trauma? How so? - Do you feel that you have been given enough training on how to manage your own emotions after dealing with other people’s trauma daily?   Q7. Compassion fatigue is a common feature of secondary trauma – this is a type of emotional, physical, and psychological burnout that results from helping other people. If this resonates with you, can you describe a time when your research has left you feeling emotionally drained or unable to empathise with others?  **4. Resilience and Coping**  4.1. What is resilience?  Q8. Moving now to a much lighter note, can you describe what does resilience mean to you?  Q9. Using your definition, how would you describe ***your*** resilience?   - What do you think has contributed/acted as a barrier to your level of resilience? (extra/prompt).   4.2. How are they coping presently?  Q10. Do you feel that your work impacts your personal life in any way, and how effectively do you think you are able to separate your work-life from your home-life?   - Some people have stated that they experience alterations in their views – self, others, and the world more generally. Do you relate to this, and if so can you share how your views have changed? - How connected do you feel to your loved ones (e.g., family, friends, relationships etc.)? - Do you ever feel isolated or unsupported in your role because of the nature of work you do? Family or Workmates - Is there anyone that you talk to specifically about the nature of your work and the impact it has on you? Is there anyone you would like to but feel you can’t? If so, who and why? (extra/prompt)   Q11. Please describe your way(s) of coping with research-related work stress or distress (hobbies, spirituality, therapy…)   - To what extent is this helping your emotional wellbeing? (extra/prompt)   **5. Improvements**  5.1. Self-improvement  Q12. Is there anything that you are not yet doing that you believe could be beneficial to your overall wellbeing?  5.2. External support  Q13. Now directing the focus more on the organisation, you work for what support mechanisms does your employer have in place to assist you in being aware of the potential dangers of secondary trauma, and are they helping you cope with its associated dangers in any way?   1. Have you utilised them before? (extra/prompt) 2. How useful were they? (extra/prompt)   Q14. If you could suggest 3 changes that could be implemented at your workplace to improve the emotional safety, coping and resilience of violence and abuse researchers, what would they be?  **6. Any Further Input**  Is there anything you think we have not covered that you think we should have covered, or that you think is important for us to discuss?  **7. Closing statements**  We have now finished with the interview. Thank you so much for your time and for your responses. Do you have any further questions about the study before we conclude? |
| --- |
