# Supplementary material for: Improving Emotional Safety, Coping, and Resilience Among Women Conducting Research on Sexual and Domestic Violence and Abuse
Source: J Interpers Violence. 2023 Oct 24;39(5-6):1327–50. doi: 10.1177/08862605231207617 (PMC10858617; doi:10.1177/08862605231207617)
Supplement: sj-docx-3-jiv-10.1177_08862605231207617 – Supplemental material for Improving Emotional Safety, Coping, and Resilience Among Women Conducting Research on Sexual and Domestic Violence and Abuse [file sj-docx-3-jiv-10.1177_08862605231207617.docx]

**Appendix C. Thematic analysis coding framework**

| **Key themes** | **Description of theme** |
| --- | --- |
| **Expertise (perceived)** | The participants’ subjective perception of their level of expertise. |
| **General feelings towards work** | The positive or negative feelings expressed by the researcher regarding their own violence research. |
| **Impact of COVID** | The ways in which the pandemic impacted participants’ work practices, style, and wellbeing |
| **Organisational support** | The presence, or lack of, training and peer or supervisory support. |
| **Participants’ characteristics** | Either their personal history with violence/abuse, or characteristics that may increase/decrease their vulnerability to violence victimization. |
| **Personal coping mechanisms** | Personal ways of dealing with feelings of secondary trauma:  Destructive behaviors  Social support  Hobbies  Mindfulness, therapy, and spirituality  Self-care |
| **Resilience** | Participants’ self-assessed degree of resilience. |
| **Secondary trauma** | Specific symptoms of secondary trauma:  Behavioural  Cognitive and altered beliefs  Emotional and compassion fatigue  Interpersonal  Physical |
| **Workplace stress** | Stressors encountered by researchers in the workplace:  Emotional impacts  Relationship with others  Lack of balance  Burn out  Cognitive and physical effects  Workload and caseload |
| **Suggestions for organizational improvement** | Participant’s opinions on how their organization could improve the response to secondary trauma in researchers. |
